# Maternal and perinatal outcomes in women with eclampsia by mode of delivery at Riley mother baby hospital: a longitudinal case-series study

**DOI:** 10.1186/s12884-021-03875-6

**Published:** 2021-06-24

**Authors:** Koech Irene, Poli Philippe Amubuomombe, Richard Mogeni, Cheruiyot Andrew, Ann Mwangi, Orang’o Elkanah Omenge

**Affiliations:** 1 Reproductive Health, Moi Teaching& Referral Hospital, PO Box 3-30100, Nandi road, Eldoret, Kenya; 2 Department of Reproductive Health, Moi University School of Medicine, P. O. Box 4606-30100, Eldoret, Kenya; 3 Department of Behavioural Sciences, Moi University School of Medicine, P.O. Box 4606-30100, Eldoret, Kenya

**Keywords:** Maternal and perinatal outcomes, Eclampsia, Mode of delivery, Resource-limited settings

## Abstract

**Background:**

Eclampsia, considered as serious complication of preeclampsia, remains a life-threatening condition among pregnant women. It accounts for 12% of maternal deaths and 16–31% of perinatal deaths worldwide. Most deaths from eclampsia occurred in resource-limited settings of sub-Saharan Africa. This study was performed to determine the optimum mode of delivery, as well as factors associated with the mode of delivery, in women admitted with eclampsia at Riley Mother and Baby Hospital.

**Methods:**

This was a hospital-based longitudinal case-series study conducted at the largest and busiest obstetric unit of the tertiary hospital of western Kenya. Maternal and perinatal variables, such as age, parity, medications, initiation of labour, mode of delivery, admission to the intensive care unit, admission to the newborn care unit, organ injuries, and mortality, were analysed using the Statistical Package for the Social Sciences software version 20.0. Quantitative data were described using frequencies and percentages. The significance of the obtained results was judged at the 5% level. The chi-square test was used for categorical variables, and Fisher’s exact test or the Monte Carlo correction was used for correction of the chi-square test when more than 20% of the cells had an expected count of less than 5.

**Results:**

During the study period, 53 patients diagnosed with eclampsia were treated and followed up to 6 weeks postpartum. There was zero maternal mortality; however, perinatal mortality was reported in 9.4%. Parity was statistically associated with an increased odds of adverse perinatal outcomes (*p* = 0.004, OR = 9.1, 95% CI = 2.0–40.8) and caesarean delivery (*p* = 0.020, OR = 4.7, 95% CI = 1.3–17.1). In addition, the induction of labour decreased the risk of adverse outcomes (*p* = 0.232, OR = 0.3, 95% CI = 0.1–2.0).

**Conclusion:**

There is no benefit of emergency caesarean section for women with eclampsia. This study showed that induction of labour and vaginal delivery can be successfully achieved in pregnant women with eclampsia. Maternal and perinatal mortality from eclampsia can be prevented through prompt and effective care.

**Supplementary Information:**

The online version contains supplementary material available at 10.1186/s12884-021-03875-6.

## Background

Eclampsia is among the most common causes of maternal and perinatal mortality and morbidities. It refers to the occurrence of one or more seizures and/or unexplained coma before, during and after birth irrespective of one’s history of hypertensive disorders, including preeclampsia [1–3]. The condition has been recognized and described for years despite the general lack of understanding of the aetiology of the disease. To date, eclampsia is considered as complication of severe preeclampsia because the majority of affected pregnant women (approximately 84%) have hypertensive disorders, and 16% of them have normal blood pressures [3]. In low- and middle-income countries (LMICs), sub-Saharan Africa included nearly 17.9% of women with eclampsia and other hypertensive disorder-related complications during pregnancy (stroke, coagulopathies). Furthermore, eclampsia is among the leading causes of intensive care unit (ICU) admission [4, 5]. The disease has been extensively studied [1–6], and the evidence has recommended prompt delivery to reduce maternal and perinatal mortality and morbidity [6]. However, little has been done regarding the maternal and perinatal outcomes by mode of delivery in the particular context of resource-limited settings. Therefore, this study was performed to determine the mode of delivery in women with eclampsia in the largest tertiary hospital of western Kenya.

## Methods

This was a hospital-based longitudinal case-series study, which consecutively recruited pregnant and postpartum women diagnosed with eclampsia in a one-year period at the Riley Mother and Baby Hospital (RMBH). The RMBH is located within Moi Teaching and Referral Hospital, the second largest national hospital of Kenya. The inclusion criteria were any women diagnosed with eclampsia during pregnancy and postpartum period between June 2019 and June 2020. All patients enrolled into the study were followed up to 6 weeks postpartum.

### Diagnostic criteria

The diagnostic criteria were onset of convulsions and/or unexplained loss of consciousness in pregnant and postpartum mothers with a negative malaria test and no previous history of convulsive disorders prior to pregnancy.

The test for protein in urine used dipstick for all participants followed by urinalysis to rule out urinary tract infection. Proteinuria and elevated blood pressure (BPs) were not diagnostic criteria for eclampsia, considering that women with negative proteinuria and normal blood pressure can develop eclampsia. By using the dipstick, patients’ results were classified as follows: trace =0.1 g/l, 1 plus (1+) = 0.3 g/l, 2 plus (2+) =1.0 g/l, 3 plus (3+) = 3.0 g/l, and 4 plus (4+) =10.0 g/l. Patients’ BPs were recorded as < 140/90 mmHg, considered as normal and ≥ 140/90 mmHg as per definition of hypertension in pregnancy after the 20th week of gestational. The initial BPs before any administration of drugs during convulsions was considered as accurate.

In addition, conditions such meningitis, hypoglycaemic coma and/or alcoholic coma, which could confuse the diagnosis of eclampsia, were ruled out through the patients’ history, physical examination and laboratory investigations.

### Clinical presentation and data collection

In majority of cases, eclampsia is preceded by features of preeclampsia. Therefore, the variables collected included symptoms of the disease such as convulsion, epigastric pain, dyspnoea, coma, palpitation, lower limb oedema, headache, blurred vision, and elevated blood pressure.

The patients with a confirmed diagnosis of eclampsia were interviewed. Data on maternal demographics, clinical characteristics, outcomes and perinatal outcomes were collected. Maternal demographic included maternal age, education level, marital status, occupation, geographic residence location, and health insurance coverage.

Clinical characteristics included last menstrual period to determine the gestational age, history of pregnancy loss, previous history of eclampsia and/ or preeclampsia, and parity. Based on their parity, patients were classified as nulliparous, multiparous, and grand multiparous. In this study, multiparous was defined as a patient who has had 4 births or less (live or stillbirth), at ≥28 weeks gestational, whereas grand multiparous was defined as a patient who has had ≥5 births at ≥28 weeks gestational regardless the outcome.

In addition, variables on comorbidities were also collected. These included diabetes mellitus, chronic hypertension, hyperthyroid disease, renal disease, coagulopathy, human immunodeficiency virus/acquired immunodeficiency syndrome, venous thromboembolism, anaemia, malnutrition, and mental illness), and treatment prior to or during pregnancy.

The study was interested in medication during antenatal care period, mostly antihypertensive for those who could have chronic hypertension or preeclampsia, and the treatment the patients received during eclamptic seizures. These included magnesium sulfate (MgSO4); antihypertensive mainly labetalol or hydralazine, and nifedipine (adalate); corticosteroids for foetal lungs maturity. The full regimen of magnesium sulfate was given to all patients, which included a loading dose of 4 g, followed by 1 g hourly for 24-h maintenance therapy following delivery. The MgSO4 toxicity was closely monitor through patients’ vital signs (oxygen saturation, deep tendon reflexes, and level of consciousness) and serum magnesium levels.

Intravenous labetalol was given, 20 mg for all patients with blood pressures (BPs) of ≥160/110 mmHg or the mean arterial pressure (MAP) of ≥125 mmHg every 20 min until the BPs dropped down to < 150/ 100 mmHg, then labetalol was shifted to nifedipine 20 mg three times daily till normalization of BPs. All women with gestational age < 34 weeks were given at least single dose of 12 mg of dexamethasone intramuscular, before induction of labour or C-section as per RMBH protocol.

For patients who were transferred from peripheral facilities, the information regarding the medications was recorded as well. Following the national guidelines, those patients received at least a loading dose of magnesium sulfate either 10 g intramuscular (5 g in each buttock) or 4 mg intravenous over 20 min, and 20 mg of nifedipine before being transferred to RMBH for further management. The 24-h maintenance therapy of magnesium sulfate was completed at RMBH.

During the course of treatment, the study grouped the patients in 2, those who received anticonvulsant (magnesium sulfate) only, and those who received multiple drugs including antihypertensive, anticonvulsants, and corticosteroids. Patients who were admitted to intensive care unit also received either diazepam or ketamine according to Moi Teaching and Referral Hospital ICU protocol.

The variables related to antenatal care attendance and facility attended, mode of admission, mode of delivery (vaginal or caesarean section delivery, indication of C-section was recorded), and mode of initiation of labour were recorded.

Regarding antenatal care visit, the study identified 2 groups: one consisted of pregnant mothers who attended antenatal clinic regardless of the frequency, and another group consisted of pregnant women who did not attend ANC visit. Those who attended antenatal care visit were grouped in 6 levels of national care grouping system, including health dispensaries (level 2), health centre (level 3), sub-county (level 4), which represent primary care level. County hospitals (level 5), known as district hospitals, representing secondary care level; and national teaching and referral hospitals (level 6), represent the tertiary care level [7]. Private facilities were included whereas community services (level 1) were excluded.

Induction of labour (IOL) was defined as the process of artificially stimulating the uterus to start labour. The study used different methods of IOL, which included misoprostol 25 micrograms either sub-lingual or vaginal, cervical insertion of Foley catheter size 16 or 18 ballooned with 80 ml normal saline, and combination of misoprostol 25 micrograms sub-lingual and cervical insertion of Foley catheter. These three methods of induction of labour are approved by the RMBH protocol. The IOL involved exclusively pregnant women admitted with a confirmed diagnosis of eclampsia, but without established labour. The decision of induction was influenced by a reassuring foetal status on electronic foetal monitor (cardiotocogram), and maternal status. However, there were patients referred at immediate postpartum, either for recurrent eclamptic seizures or prematurity. For patients who delivered at RMBH through C-section, the presence of paediatrician was documented as per RMBH protocol.

### Maternal and perinatal outcomes

Maternal outcomes of interest included the occurrence of ≥1 of the following complications: stroke, acute kidney injuries (AKI), HELLP syndrome (Haemolysis, Elevated Liver enzymes, and Low Platelet), heart failure, pulmonary oedema, coagulopathy, ICU admission, and mortality or recovery within 6 weeks postdelivery.

The haemorrhagic stroke, life-threatening complication of preeclampsia and eclampsia was ruled out in few patients who had recurrent eclamptic seizures or status eclampticus, neurological deficits, and prolonged coma. The CT scan was used to diagnose the haemorrhagic stroke. The tests for renal function were performed to all participants. The diagnosis of AKI was based on the serum creatinine > 80 mmol/l. In addition, laboratory tests such full haemogram, abnormal peripheral blood smear (intravascular haemolysis), increased serum bilirubin (≥ 20.5 μmol/L or ≥ 1.2 mg/100 mL) and elevated LDH levels (> 600 units/L (U/L) were used for the diagnosis of HELLP syndrome. However, the nadir platelet count alone during the course of the disease was also considered as diagnosis of HELLP syndrome. Therefore, patients classified into complete and incomplete HELLP syndrome [8], based on laboratory results described above.

Perinatal outcomes of interest were defined by the occurrence ≥1 of the following complications: stillbirths (fresh or macerated), Apgar score < 7 at five minutes, newborn unit (NBU) admission, and mortality or recovery within 7 days following delivery. The study was interested on the 5-min Apgar score to determine newborn’s neurologic outcome. Immediately after delivery, either vaginally or through C-section, all newborns were appropriately assessed by the primary nurse or midwife and paediatrician to determine the need for admission to nursery. Every baby born from eclamptic mother outside RMBH was appropriately assessed on admission to determine whether nursery admission was required. In addition, newborns were classified in 4 groups based on their birth weights: < 1000 g, 1000 g- 1500 g, 1600 g- 2400 g, and 2500 g- 400 g.

The mode of delivery was defined as the dependent variable. The primary outcome was defined by the occurrence of ≥1 adverse maternal and perinatal outcomes indicated above. The secondary outcome was defined by recovery or persistence of maternal complications 6 weeks postdelivery and 7 days following birth for newborns.

Authors received ethical approval to conduct the study. All patients enrolled to the study provided a written informed consent. Six weeks’ postdelivery period of follow up was completed for all patients. Follow up was conducted during hospitalization and after discharge from the ward for each patient. During follow up, the study was particularly interested on either improvement or worsening condition of each patient vital signs, laboratory tests, and the status of the newborn. The appointment schedules were given they of discharging the patient from ward, on basis of the treating doctors and captured by the research assistants.

### Data analysis

The data were analysed using IBM SPSS software package version 20.0. (Armonk, NY: IBM Corp). Categorical data are described as frequencies and percentages, while continuous data are described as ranges, means, standard deviations and medians. The Kolmogorov-Smirnov test was used to verify the normality of the distribution of quantitative data. The significance of the obtained results was judged at the 5% level. The chi-square test was used for categorical variables to compare differences between groups based on their outcomes. Monte Carlo correction was used to improve the accuracy of the *p*-value for the chi-square analysis when more than 20% of the cells had an expected count of less than 5. The odds ratio and 95% confidence interval (CI) for maternal adverse outcomes were calculated using logistic regression.

## Results

During the study period (June 2019 to June 2020), 53 pregnant and postpartum women were admitted with a diagnosis of eclampsia. Maternal socio-demographics can be seen in Supplementary Table 1.1, and clinical characteristics can be seen in Supplementary Table 2.1. Maternal blood pressures and laboratory results are see in supplementary Table 2.2. Supplementary Table 2.3 represents the management of mothers admitted with eclampsia during the study period.

### Maternal and perinatal outcomes

Table 1 represents maternal and perinatal outcomes. There was no maternal death; however, 5.7% of mothers were admitted to the ICU. Additionally, 20.8% of patients had true HELLP) syndrome while 11.3% had incomplete HELLP syndrome; 15.1% others had acute kidney injury (AKI); and 1.9% developed stroke (Table 1). All patients recovered from the disease within 6 weeks postdelivery of follow up, including follow up after discharge from hospital.

**Table 1:** Maternal and perinatal outcomes

Outcome	No.	%
**Maternal outcome**		
PPH	0	0.0
Death	0	0.0
ICU admission	3	5.7
Acute kidney injury (AKI)	8	15.1
Incomplete HELLP syndrome	6	11.3
Complete HELLP syndrome	11	20.8
Haemorrhagic stroke	1	1.9
Recovery from the disease	53	100.0
Sequelae	0	0.0
On follow up 6 weeks postdelivery	0	0.0
**Perinatal outcome**		
**Death**		
Yes	5	9.4
No	48	90.6
**Birth weight**		
< 1000 g	4	7.5
1000 g–1500 g	17	32.1
1600 g–2400 g	24	45.3
2500 g–4000 g	8	15.1
**NBU admission**		
Yes	18	34.0
No	35	66.0
**Stillbirth**		
NA	48	90.6
Fresh	3	5.7
Macerated	2	3.8
**Score ≥ 7**		
NA	5	7.5
Yes	37	69.8
No	11	20.8

*PPH* postpartum haemorrhage, *ICU* intensive care unit, *NBU* newborn unit, *NA* not applicable, *HELLP* elevated liver enzymes and low platelet

Regarding perinatal outcomes, 34.0% of newborns were admitted to the newborn unit (NBU). Mortality was reported in 9.4% of cases, and 7.5% were fresh stillbirths. The majority of newborns (73.6%) were born with a good Apgar score of > 7 at 5 min, whereas only 20.8% of newborns had an Apgar score < 7 at 5 min. The score was not applied for 7.5% of stillbirths.

The socio-demographic and clinical characteristics were analysed to determine factors that could be associated with the mode of delivery in pregnant women with eclampsia. The results reported in Table 2 show that socio-demographic factors, including maternal age, occupation, health insurance coverage, and geographic location of residence, were not found to be associated with the mode of delivery, even after adjustments were made for confounders. However, low maternal education level increased the odds of caesarean delivery 8-fold (*p* = 0.029. 95% CI = 1.2–51.5).

**Table 2:** Maternal socio-demographic characteristics and mode of delivery, using Fisher Exact test or chi-square test

Variables	Mode of delivery				***p-***value	OR	***p-***value	95%CI
	Vagina (***n*** = 24)		Caesarean (***n*** = 29)					
	No.	%	No.	%				
**Maternal age group**								
< 20 years^#^	3	12.5	7	24.1	^MC^*p =* 0.466	–	–	–
20–34 years	17	70.8	16	55.2		0.4	0.240	0.1–1.8
35–44 years	4	16.7	6	20.7		0.6	0.640	0.1–4.1
**Education level**								
Low education level	3	12.5	9	31.0	0.068	8.0	0.029*	1.2–51.5
Secondary	13	54.2	17	58.6		3.5	0.105	0.8–15.8
Tertiary^#^	8	33.3	3	10.3		–	–	–
**Marital status**								
Married^#^	17	70.8	13	44.8	0.057	–	–	–
Single	7	29.2	16	55.2		2.9	0.061	0.9–9.4
**Health insurance**								
Yes^#^	13	54.2	12	41.4	0.353	–	–	–
No	11	45.8	17	58.6		1.7	0.355	0.6–4.9
Employees®	19	79.2	22	75.9	0.775	–	–	–
Unemployed	5	20.8	7	24.1		1.2	0.775	0.3–4.4
**Residence location**								
Rural	21	87.5	26	89.7	^FE^*p =* 1.000	1.2	0.8	0.2–6.8
Urban^#^	3	12.5	3	10.3		–	–	–

*MC* **Monte Carlo**, *FE* **Fisher Exact**

*p p* value for association between different categories

*OR* Odds ratio, *CI* Confidence interval, *LL* Lower limit, *UL* Upper Limit

*: Statistically significant at *p* ≤ 0.05

#: no *p-*value

In addition, clinical factors such as previous history of eclampsia, symptoms of the disease, parity, history of pregnancy loss, gestational age, and antenatal care attendance were not significantly associated with the mode of delivery, even after adjustments were made for confounders, as shown in Table 3. In contrast, the method of the initiation of labour was statistically associated with the mode of delivery, as reported in Table 4.

**Table 3:** Mode of delivery and clinical characteristics, using Fisher Exact test or chi-square test

Clinical Characteristics	Mode of delivery				***p-***value	OR	***p-***value	95%CI
	Vaginal (***n =*** 24)		Caesarean (***n =*** 29)					
	No.	%	No.	%				
**Medical History**								
None^#^	23	95.8	29	100.0	^FE^*p =* 0.453	–	–	–
Chronic hypertension	1	4.2	0	0.0		–	–	–
**Symptoms**								
Epigastric pain	9	37.5	13	44.8	0.590	1.4	0.590	0.4–4.1
Coma	4	16.7	1	3.4	^FE^*p =* 0.164	0.2	0.136	0.0–1.7
Lower Limbs oedema	10	41.7	8	27.6	0.281	0.5	0.284	0.2–1.7
Headache	16	66.7	18	62.1	0.728	0.8	0.728	0.3–2.5
Blurred vision	1	4.2	5	17.2	^FE^*p =* 0.204	4.8	0.167	0.5–44.2
Convulsions	20	83.3	28	96.6	^FE^*p =* 0.164	5.6	0.136	0.6–53.9
**Obstetric history**								
Nulliparous	11	45.8	12	41.4	0.745	0.8	0.745	0.3–2.5
Multiparous (≤4 births)	9	37.5	16	55.2	0.200	2.1	0.202	0.7–6.2
Grand-Multiparous (≥5b)	4	16.7	1	3.4	^FE^*p =* 0.164	0.2	0.136	0.0–1.7
**History of pregnancy loss**								
Yes	3	12.5	5	17.2	^FE^*p =* 0.715	1.5	0.633	0.3–6.8
No^#^	21	87.5	24	82.8		–	–	–
**Gestational age**								
≤ 28 week	4	16.7	0	0.0	^MC^*p =* 0.075	–	–	–
28–33 weeks	7	29.2	14	48.3		1.0	0.853	0.2–4.4
34–37 weeks	8	33.3	5	17.2		0.3	0.141	0.0–1.5
38–40 weeks ^#^	3	12.5	7	24.1		–	–	–
LMP unknown	2	8.3	3	10.3		0.6	0.699	0.1–6.1
**Gestational period at time of enrollment**								
Intrapartum ^#^	1	4.2	0	0.0	^FE^*p =* 0.453	–	–	–
Postpartum	23	95.8	29	100.0		–	–	–
**ANC visit attendance**								
Yes	22	91.7	27	93.1	^FE^*p =* 1.000	1.2	0.844	0.2–9.4
No#	2	8.3	2	6.9		–	–	–

*ANC* antenatal care

**Table 4:** Mode of delivery and maternal management, using Fisher Exact test or chi-square test

Variables	Mode of delivery				***p-***value	OR	***p-***value	95%CI
	Vaginal (***n =*** 24)		Caesarean (***n =*** 29)					
	No.	%	No.	%				
**Facility of ANC visit**								
No ANC visit^#^	2	8.3	2	6.9	^MC^*p =* 0.243	–	–	–
Teaching &Referral Hosp	1	4.2	0	0.0		–	–	–
County referral Hospital	4	16.7	0	0.0		–	–	–
Sub-county Hospital	3	12.5	3	10.3		1.0	1.000	0.1–12.6
Health centre	9	37.5	17	58.6		1.9	0.557	0.2–15.7
Private Hospital	1	4.2	1	3.4		1.0	1.000	0.0–29.8
Dispensary	4	16.7	6	20.7		1.5	0.733	0.1–15.5
**Mode of admission**								
From home^#^	12	50.0	12	41.4	^FE^*p =* 0.587	–	–	–
Transferred	12	50.0	17	58.6		1.4	0.531	0.5–4.2
**Treatment of HTN**								
Yes	5	20.8	5	17.2	^FE^*p =* 1.000	0.8	0.740	0.2–3.1
No^#^	19	79.2	24	82.8		–	–	–
**Medications**					^MC^*p =* 1.000			
Anticonvulsants^#^	4	16.7	4	13.8		–	–	–
Multiples drugs	20	83.3	25	86.2		1.3	0.771	0.3–5.6
**Labour initiation**								
None^#^	0	0.0	17	58.6	^MC^p < 0.001^*^			
**Spontaneous**	15	62.5	12	41.4				
**Induction:**								
Cytotec	4	16.7	0	0.0		–	–	–
Foley catheter	1	4.2	0	0.0				
Foley catheter+ Cytotec	4	16.7	0	0.0				

*ANC* antenatal care

Maternal blood pressures and laboratory test results were not associated with mode of delivery, as indicated in Table 5. Furthermore, as shown in Table 6, maternal complications such as haemorrhagic stroke, HELLP syndrome, and AKI were not associated with the mode of delivery.

**Table 5:** Mode of delivery and maternal BPs and laboratory results, using Fisher Exact test or chi-square test

Variables	Mode of delivery				***p-***value	OR	***p-***value	95%CI
	Vaginal (***n =*** 24)		Caesarean (***n =*** 29)					
	No.	%	No.	%				
**Blood pressures**								
< 140/90 mmHg^#^	2	8.3	5	17.2	^FE^*p =* 0.436	–	–	–
≥ 140/90 mmHg	22	91.7	24	82.8		0.4	0.350	0.077–2.483
**Proteinuria**								
Trace^#^	1	4.2	6	20.7	^MC^*p =* 0.133	–	–	–
1+	6	25.0	2	6.9		6.0	0.165	0.478–75.34
3+	13	54.2	17	58.6		0.3	0.309	0.040–2.769
4+	4	16.7	4	13.8		1.3	0.737	0.274–6.240
**Platelet**								
≥ 150.10^9^/l	19	79.2	23	79.3	^MC^*p =* 0.694	0.6	0.585	0.100–3.672
≤ 100.10^9^/l^#^	2	8.3	4	13.8		–	–	–
≤ 50.10^9^/l	3	12.5	2	6.9		0.3	0.383	0.028–.028
**Peripheral blood smear**								
Normal^#^	21	87.5	29	100	^FE^*p =* 0.086	–	–	–
Abnormal	3	12.5	0	0.0		–	–	–
**AST/ALT**								
< 70 IU/l^#^	21	87.5	26	89.7	^FE^*p =* 1.000	**–**	–	–
≥ 70 IU/l	3	12.5	3	10.3		1.2	0.806	0.226–6.781
**Serum bilirubin**								
Normal^#^	22	91.7	27	93.1	^FE^*p =* 1.000	–	–	–
Increased	2	8.3	2	6.9		1.2	0.844	0.160–9.431
**LDH**								
< 600 IU/l^#^	22	91.7	27	93.1	^FE^*p =* 1.000	–	–	–
≥ 600 IU/l	2	8.3	2	6.9		0.8	0.844	0.106–6.261
**Creatinine**								
≤ 80 mmol/l^#^	19	79.2	26	89.7	^FE^*p =* 0.444	–	–	–
> 80 mmol/l	5	20.8	3	10.3		2.3	0.297	0.485–10.73

*AST* aspartate transaminase, *ALT* alanine transaminase, *LDH* L-lactate dehydrogenase

**Table 6:** Mode of delivery and maternal outcomes

Variables	Mode of delivery				^**FE**^p	OR	***p-***value	95%CI
	Vaginal (***n =*** 24)		Caesarean (***n =*** 29)					
	No.	%	No.	%				
**Maternal outcome**								
ICU admission	0	0.0	3	10.3	0.242	–	–	–
Recovery^#^	24	100.0	29	100	–	–	–	–
**Complications**								
Kidney injury	5	20.8	3	10.3	0.444	0.4	0.297	0.1–2.1
Incomplete HELLP synd	3	12.5	3	10.3	1.000	1.2	0.806	0.2–6.9
Stroke	0	0.0	1	3.4	1.000	–	–	–
True HELLP syndrome	5	20.8	6	20.7	1.000	0.9	0.990	0.3–3.8

In Table 7, factors such maternal history of chronic hypertension, symptoms, history of pregnancy loss, antenatal care attendance, and birth weights were not associated with the risk of newborn admission to the NBU, even after adjustments were made for confounders. In contrast, parity was significantly associated with the risk of newborn admission to the NBU. After adjustments were made for confounders, infants from multiparous women had 7.6-fold increased odds of being admitted to the NBU (*p* = 0.003, 95% CI = 2.0–28.6).

**Table 7:** NBU admission and maternal clinical characteristics and birth weight, using Fisher Exact test or chi-square test

Clinical Characteristics	NBU admission				***p-***value	OR	***p-***value	95%CI
	No (***n =*** 35)		Yes (***n*** = 18)					
	No.	%	No.	%				
**Medical History**								
None^#^	34	97.1	18	100.0	^FE^*p =* 1.000	–	–	–
Chronic HTN	1	2.9	0	0.0		–	–	–
**Clinical presentation or complaint**								
Epigastric pain	15	42.9	7	38.9	0.781	0.8	0.781	0.3–2.7
Coma	4	11.4	1	5.6	^FE^*p =* 0.651	0.5	0.498	0.0–4.4
Lower limbs oedema	12	34.3	6	33.3	0.945	0.9	0.945	0.3–3.2
Headache	21	60.0	13	72.2	0.380	1.7	0.382	0.5–5.9
Blurred vision	4	11.4	2	11.1	^FE^*p =* 1.000	0.9	0.972	0.2–5.9
Convulsions	31	88.6	17	94.4	^FE^*p =* 0.651	0.5	0.498	0.0–4.4
**Obstetric history**								
Nulliparous	19	54.3	4	22.2	0.026^*^	–	–	–
Multiparous (≤ 4 births)	11	31.4	14	77.8	0.001^*^	6.0	0.008^*^	1.6–23.0
Grand-Multiparous (≥5 b)	5	14.3	0	0.0	^FE^*p =* 0.153	–	–	–
**History of pregnancy loss**								
Yes	4	11.4	4	22.2	^FE^*p =* 0.421	0.5	0.306	0.1–2.1
No^#^	31	88.6	14	77.8		–	–	–
**Gestational period at time of enrollment**								
Intrapartum ^#^	1	2.9	0	0.0	^FE^*p =* 1.000	–	–	–
Postpartum	34	97.1	18	100.0		–	–	–
**ANC visit attendance**								
Yes	31	88.6	18	100.0	^FE^*p =* 0..287	–	–	–
No^#^	4	11.4	0	0.0		–	–	–
**Birth weight**					^MC^*p =* 0.364			
< 1000 g	1	2.9	3	16.7		5.0	0.239	0.3–72.8
1000 g–1500 g	12	34.3	5	27.8		0.7	0.687	0.1–4.1
1600 g–2400 g	17	48.6	7	38.9		0.7	0.661	0.1–3.7
2500 g–4000 g^#^	5	14.3	3	16.7		–	–	–

In addition, Table 8 represents NBU and maternal management during the course of the disease. The mode of delivery, indication of C-section, and method of labour initiation were significantly associated with the risk of admission to the NBU. Similarly, infants born through caesarean section had a 4.7-fold increase of being admitted to the NBU (*p* = 0.020, 95% CI = 1.3–17.1). Eclampsia as an indication of C-section increased the risk of admission to the NBU 4.3-fold (*p* = 0.031, 95% CI = 1.1–16.1). Additionally, initiation of labour, either spontaneous or induced were not statistically associated with increased risk of admission to the NBU (see Table 8).

**Table 8:** NBU admission and maternal management, using Fisher Exact test or chi-square test

Variables	NBU admission				***p-***value	OR	***p-***value	95%CI
	No (***n =*** 35)		Yes (***n =*** 18)					
	No.	%	No.	%				
**Facility of ANC visit**								
None^#^	4	11.4	0	0.0	^MC^*p =* 0.354	–	–	–
Teaching &Referral Hosp	1	2.9	0	0.0		–	–	–
County referral Hosp	2	5.7	2	11.1		–	–	–
Sub-county Hosp	5	14.3	1	5.6		–	–	–
Health centre	14	40.0	12	66.7		–	–	–
Private Hosp	1	2.9	1	5.6		–	–	–
Dispensary	8	22.9	2	11.1		–	–	–
**Mode of admission**								
From home^#^	15	42.9	9	50.0	0.621	–	–	–
Transferred	20	57.1	9	50.0		0.8	0.621	0.2–2.3
**Treatment of HTN**								
Yes	7	20.0	3	16.7	^FE^*p =* 1.000	0.8	0.769	0.2–3.6
No^#^	28	80.0	15	83.3		–	–	–
**Medications**					^MC^*p =* 0.701			
Anticonvulsants^#^	6	17.1	2	11.1		–	–	–
Multiples drugs	29	82.9	16	88.9		1.655	0.564	0.3–9.2
**Mode of delivery**								
Vaginal^#^	20	57.1	4	22.2	0.016^*^	–	–	–
Caesarean	15	42.9	14	77.8		4.7	0.020^*^	1.3–17.1
**Indication of C-section**								
N/A^#^	20	57.1	4	22.2	^MC^*p=* 0.012^*^	–	–	–
Eclampsia	14	40.0	12	66.7		4.3	0.031*	1.1–16.1
NRFS	0	0.0	2	11.1		0.9	–	–
Arrested disorders	1	2.9	0	0.0		–	–	–
**Labour initiation**								
**N/A**(C-section)	8	22.9	9	50.0	MC*p =* 0.028*	3.9	0.036*	1.1–14.7
**Spontaneou**s^#^	21	60.0	6	33.3		–	–	–
**Induction**:								0.9–120.5
Cytotec	1	2.9	3	16.7		10.5	0.059	–
Cytotec+ F. catheter	1	2.9	0	0.0		–	–	
Foley catheter	4	11.4	0			–	–	
**Spontaneous vs induction of labour**:								
Spontaneous^#^	21	60.0	6	33.3	0.066	–	–	–
Other labour induction	14	40.0	12	66.7		3.0	0.066	0.9–9.9

*HTN* hypertension, *C-section* caesarean section, *N/A* not applicable, *NRFS* non-reassuring foetal status

Birth weights and perinatal deaths were not statistically associated with the mode of delivery, as shown in Table 9. Similarly, perinatal deaths were not associated with birth weights, as indicated in Table 10. Finally, the multivariate logistic regression showed that parity, especially multiparty, increased the risk of perinatal adverse outcomes (*p* = 0.004*, OR = 9.1, 95% CI = 2.0–40.8). Eclampsia as indication for C-section was not found to increase the risk of adverse perinatal outcome, as show in Table 11.

**Table 9:** Mode of delivery, birth weights, and perinatal deaths, using Fisher Exact test or chi-square test

Variables	Mode of delivery				***p-***value	OR	***p-***value	95%CI
	Vaginal (***n =*** 24)		Caesarean (***n =*** 29)					
	No.	%	No.	%				
**Perinatal death**								
Yes	4	16.7	1	3.4	^FE^*p =* 0.164	0.2	0.136	0.0–1.7
No^#^	20	83.3	28	96.6		–	–	–
**Birth weights**								
< 1000 g	4	16.7	0	0.0	^MC^*p =* 0.140	–	–	–
1000 g–1500 g	8	33.3	9	31.0		0.675	0.654	0.121–3.767
1600 g–2400 g	9	37.5	15	51.7		1.000	1.000	0.192–5.222
2500 g–4000 g^#^	3	12.5	5	17.2		–	–	–

**Table 10:** Perinatal death by birth weights

Variables	Perinatal death				^**MC**^p	OR	***p-***value	95%CI
	No (***n =*** 48)		Yes (***n*** = 5)					
	No.	%	No.	%				
**Birth weights**								
< 1000 g	4	8.3	0	0.0	0.896	–	–	–
1000 g–1500 g	15	31.3	2	40.0		1.0	–	–
1600 g–2400 g	21	43.8	3	60.0		1.0	–	–
2500 g–4000 g^#^	8	16.7	0	0.0		–	–	–

**Table 11:** Multivariate analysis Logistic regression for NBU admission

Variables	Sig.	OR	95% CI	
			LL	UL
Multiparity	0.004^*^	9.1	2.0	40.8
Eclampsia as indication for C-section	0.323	2.4	0.4	13.4

*LL* Lower limit, *UL* Upper Limit

## Discussion

The optimal mode of delivery in women with eclampsia remains controversial in the modern practice of obstetrics and gynaecology. In this study, the caesarean delivery rate was slightly higher than that of women who had normal vaginal delivery. This contrasts with the findings from the study done by Priti Kumari and colleagues, in which the rate of vaginal delivery was higher than that of caesarean delivery [9]. However, in most studies across the world, caesarean section delivery has been repeatedly reported to be higher in women with eclampsia [10–12]. The plausible explanation for the difference is that in most protocols, including ours, with respect to the management of eclampsia, it is recommended that delivery should occur within 12 h following seizure(s), and only pregnant women admitted in the active phase of labour or with favourable Bishop scores are allowed to progress within the 12 h if the foetal status is preserved. Additionally, the extensive use of cardiotogram (CTG) machines to monitor labour and the lack of consensus on the interpretation of the tracing have widely contributed to an increased rate of caesarean delivery [13–15]. Another aspect that could explain the increased rate of C-section delivery in eclamptic mothers is the panic attitude of midwives during seizer(s), while most of today’s midwives do not agree to monitor the labour of women with eclampsia. Experience and good exposure in the field, as well as evidence-based practice of the art of obstetrics in resource-limited settings, may curve the trends of caesarean delivery among eclamptic mothers.

Socio-demographic factors such as maternal age, education level, occupation, healthcare insurance coverage, and geographic location were not significantly associated with mode of delivery. This finding contrasts with those of previous studies in which maternal age, educational level, parity, household socioeconomic status, rural residence location, and household level of education were associated with caesarean section delivery [16, 17]. However, low maternal education increased the risk of caesarean delivery among affected women. This could be due to the easy accessibility of comprehensive emergency obstetric care, including caesarean section. We also noticed that some of these patients were operated on at county hospitals and were referred for further management of persistent seizures after delivery.

Clinical factors, including symptoms of the disease, parity, history of pregnancy loss, antenatal care attendance and facility attended for ANC, mode of admission, treatment of hypertension, and medications received during seizures, were not associated with the mode of delivery. Begun N and colleagues reported similar findings [18]. This could be explained by the good patient response to treatment. Moreover, evidence recommends that the pregnant mother with eclampsia should be stabilized before making decisions regarding delivery [19]. However, the goal of stabilizing the patient with medications is not to conserve the pregnancy but to allow for better assessment and the ability to determine the optimal and safest mode of delivery within a reasonable amount of time.

All patients from this study received magnesium sulfate as anticonvulsant. To date, magnesium sulfate (MgSO4) is first line medication and preferred anticonvulsant to stabilize pregnant mother with eclampsia [20]. It is a lifesaving drug, which slows neuromuscular conduction and decreases the central nerves system irritability by triggering cerebral vasodilation, thus reducing ischemia generated by cerebral vasospasm during an eclamptic event [20]. The advantages of using MgSO4 as drug of choice are not only limited to prevent primary and recurrent maternal seizures; but it reduces maternal and perinatal mortality and morbidity in pregnant women with pre-eclampsia and eclampsia [20–22]. It plays a role of neuroprotection by reducing the risk of cerebral palsy in grow-restricted foetus and preterm below 30 weeks of gestation [21, 22]. In Kenya, MgSO4 is an essential medication available at all different levels of care. The national guidelines for quality obstetrics and perinatal care recommend the administration of MgSO4 to all pregnant women with preeclampsia with features of severity and eclamptic women before being transferred to the high level of care [23]. In the protocol of RMBH, the administration of intravenous magnesium sulfate to all pregnant women with preeclampsia, presenting features of severity, and eclamptic mothers is mandatory. The loading dose, 4 g for 20 min, followed by maintenance intravenous dose of 1 g per hour. The treatment with MgSO4 is maintained until 24 h post-delivery or from 24-h after the last seizure. The toxicity of magnesium sulfate was monitor through patients’ vital signs (oxygen saturation, deep tendon reflexes, level of consciousness, and urinary input and output) as outlined in the national and WHO guidelines [20, 23].

In this study, majority of cases, with very elevated blood pressure, ≥160 mmHg systolic pressure and ≥ 110 mmHg diastolic pressure received both anticonvulsant and antihypertensive. This approach is described in several guidelines [23, 24]. In RMBH protocol, the first line antihypertensive severe preeclampsia and eclampsia remains intravenous labetalol 20 mg. However, other institutions’ guidelines and protocol recommend hydralazine as first line antihypertensive [25]. Hydralazine is a central vasodilator that has been used in severe hypertension and preeclampsia in pregnancy [26]. It is administered as a 5- to 10-mg dose IV or IM every 15 min up to a maximum dose of 20 mg IV or 30 mg IM [25, 26]. The onset of action hydralazine is 10 to 20 min [25, 26]. In contrast, labetalol is a nonselective, competitive beta-adrenergic and a selective, competitive alpha1-adrenergic blocking agent [26]. The recommended dose of labetalol is 20 mg over slow-infusion IV for a maximum dosage of 200 mg [23]. There are 2 potential advantages of labetalol over hydralazine, including quicker onset of action (5 min) and less risk for reflex tachycardia [25, 26]. The mechanism of action of labetalol is exerted by reduction of peripheral vascular resistance without compromising blood flow to the brain and peripheral, coronary, or renal systems [25]. These reasons explain the choice of labetalol as first-line treatment of severe preeclampsia and eclampsia at RMBH. The goal of treating acute severe hypertension in preeclampsia/ eclampsia is crucially to prevent cerebrovascular and cardiovascular events as well as maternal death. In this study, few patients who had slightly elevated blood pressure, ≤140 mmHg systolic and ≤ 90 mmHg diastolic, or nearly normal blood pressure were managed with MgSO4 only, up to 24 h post-delivery or 24 h after the last seizure. The reason of withholding antihypertensive in patients with nearly normal BPs is supported the double effective role of MgSO4: prevention and/ or control of seizures and lowering total peripheral vascular resistance (calcium antagonist).

The method of initiation of labour was significantly associated with the mode of delivery, where induction of labour (IOL) with Cytotec alone, Foley catheter alone or a combination of the Foley catheter and Cytotec showed reduced morbidity related to caesarean delivery. Pregnant women in whom labour was initiated artificially had an unfavourable cervix (poor Bishop score). However, the duration of labour was not recorded, but the absence of emergency caesarean section delivery among those women showed that vaginal delivery was achieved within a reasonable time of 12 h. In a randomized study conducted by Seal SL and colleagues on eclamptic patients, IOL was preferably the safest mode of delivery and did not increase the risk of caesarean section delivery [27]. Therefore, the authors of the current study recommend IOL in eclamptic mothers to achieve vaginal delivery, even with an unfavourable cervix. This has a reasonable implication and advantage in terms of cost and the prevention of primary caesarean section with good outcomes.

Regarding maternal outcomes, there was no maternal death reported in this study. This finding contrasts with previous studies conducted in a similar context of resource-limited settings of sub-Saharan Africa and other developing countries, where maternal mortality from eclampsia was higher [18–30]. This is the result of a clear and tight protocol for the management of the disease, as well as interdisciplinary care. For example, patients who were in critical condition after delivery were admitted to the ICU, where they were managed with a multidisciplinary team that included obstetricians, nephrologists, anaesthesiologists, neurologists and neurosurgeons, and trained nurses in intensive care. Such management approaches, as well as ICUs, are widely lacking in most resource-limited settings in sub-Saharan Africa. Therefore, improving maternal and newborn care in developing countries cannot be achieved on paper but rather through investment in health infrastructure and staff. Moreover, the World Health Organization is defined as a standard roadmap for improving maternal and newborn care in health facilities [31].

Maternal complications from the disease included HELLP syndrome, acute kidney injury, and stroke, which were not associated with the mode of delivery. To date, HELLP syndrome is classified either complete (true) or incomplete HELL syndrome [8]. The diagnostic criteria are based on Tenessee classification as described by Sibai for “true” HELL syndrome, and the Mississippi-Triple class system [8]. In the particular context of this study, patients with both complete and incomplete HELLP syndrome were involved, considering eclampsia as life-threatening disease or complication of preeclampsia.

Given the current evidence, caesarean section delivery is discouraged with HELLP syndrome and AKI due to the risk of uncontrolled bleeding and poor elimination of anaesthetic drugs [8, 32–34]. The incidence of these complications was also less frequent than that reported in other studies [1–5]. However, the reason for prompt delivery in eclampsia is to prevent serious maternal complications, including death. Townsend and colleagues state that the only cure for preeclampsia and eclampsia is the delivery of the placenta, while all other approaches merely serve to manage symptoms and stabilize the mother [6]. In line with this, the Riley Mother and Baby Hospital (RMBH) protocol has made “delivery” the gold standard for the management of eclampsia, regardless of gestational age and foetal status. Any attempt toward the conservative management of eclampsia is not permitted. To date, if several studies have agreed on non-conservative management and prompt delivery, controversy regarding the mode of delivery has persisted. However, in the current study, even if there was no maternal death, caesarean section delivery was not associated with better maternal outcomes in terms of morbidity and ICU admission. This is congruent with the findings of a randomized controlled study performed by Seal SL and colleagues, who found that C-section was not associated with better outcomes [27].

Maternal convulsive seizures are also dangerous for the foetus. However, perinatal outcomes and maternal clinical characteristics, especially previous history of eclampsia, symptoms of the disease, history of pregnancy loss, antenatal care attendance and facility attended for ANC, mode of admission, treatment of HTN, and medications during eclamptic seizures, were not associated with the risk of infants being admitted to the NBU. This is because eclampsia is associated with transient maternal hypoxic status, which has minimal transient effects on the foetus [19]. In settings where there is a CTG machine, the effects of maternal hypoxia on the foetus during seizures are shown by transient reduced variability and bradycardia for up to 20 min after maternal seizures [19]. Persistent reduced variability may be related to the effects of drugs used to control and stabilize maternal conditions and/or persistent maternal hypoxia during status eclampticus [6]. In this study, parity, mode of delivery, indication of C-section delivery, and method of initiation of labour were significantly associated with the risk of infants’ admission to the NBU. Regarding parity, Melese MF and colleagues had similar findings, especially in nulliparous and multiparous patients [35, 36]. This could be related to individual factors such as the severity of the disease, drug effects, prematurity, and/or mode of delivery. In addition, caesarean section delivery increased the risk of newborns’ admission to the nursery, especially those for whom the indication for C-section was eclampsia, whereas IOL significantly reduced the risk of NBU admission. Vaginal delivery slightly increased the rate of perinatal death. Indeed, majority of perinatal were fresh stillbirths and macerated stillbirths. The fresh stillbirths occurred in mothers who were admitted with severe foetal bradycardia, and in the doctors’ judgement, C-section delivery could not help. There was 1 fresh stillbirth from C-section where the indication was eclampsia. Surgical skills during the procedure, “difficult extraction”, and the effects of anaesthesia, prematurity were major contributors for that specific case. Therefore, caesarean section delivery is not associated with better perinatal outcomes. It should be performed for obstetric reasons, defined as inability to accomplish a vaginal delivery within a specified time, governed by maternal condition. However, due to observational nature of the study, authors acknowledge the limitations of the study, which can not be used to demonstrate causes of observed adverse outcomes.

## Conclusion

Emergency caesarean section delivery offers no benefit for maternal and perinatal outcomes in women with eclampsia. Although observational in nature, this study showed that it is possible to achieved vaginal delivery through induction of labour in eclamptic mothers with unfavourable Bishop scores. Knowing eclampsia is life-threatening disease or complications for pregnant women, this study showed that maternal mortality can be reduced or prevented with prompt and effective care.

## Limitations and recommendations

This study was conducted in one tertiary hospital, and the sample size was too small to be generalized. In addition, this was not a randomized clinical trial; therefore, the evidence that it provides can not be generalized. Further multi-centres and randomized study with a large sample size is needed to interrogate current findings.

## Supplementary Information

**Additional file 1: Table 1.1.** Socio-demographic characteristics. **Table 2.1.** Maternal clinical characteristics. **Table 2.2.** Maternal BPs and laboratory results. **Table 2.3.** Management.

**Additional file 2.**

## Data Availability

All data generated or analysed during this study are included in this published article [and its supplementary information files].

## Abbreviations

ANC: Antenatal care
ICU: Intensive care unit
IOL: Induction of labour
