# Supplementary material for: Maternal and perinatal outcomes in women with eclampsia by mode of delivery at Riley mother baby hospital: a longitudinal case-series study
Source: BMC Pregnancy Childbirth. 2021 Jun 24;21:439. doi: 10.1186/s12884-021-03875-6 (PMC8228929; doi:10.1186/s12884-021-03875-6)
Supplement: Supplementary file 1 — Additional file 1: Table 1.1. Socio-demographic characteristics. Table 2.1. Maternal clinical characteristics. Table 2.2. Maternal BPs and laboratory results. Table 2.3. Management. [file 12884_2021_3875_MOESM1_ESM.docx]

**SUPPLEMENTARY TABLES**

1. **Maternal characteristics**

**Table 1.1. Socio-demographic characteristics**

| **Variables** | **No.** | **%** |
| --- | --- | --- |
| **Maternal age group** |  |  |
| <20 years | 10 | 18.9 |
| 20-34 years | 33 | 62.3 |
| 35-44 years | 10 | 18.9 |
| **Education level** |  |  |
| Low ed. level | 12 | 22.6 |
| Secondary | 30 | 56.6 |
| Tertiary | 11 | 20.8 |
| **Marital status** |  |  |
| Married | 30 | 56.6 |
| Single | 23 | 43.4 |
| **Health insurance** |  |  |
| Yes | 25 | 47.2 |
| No | 28 | 52.8 |
| **Profession** |  |  |
| Housewives | 32 | 60.4 |
| Students | 9 | 17.0 |
| Self-employee | 10 | 18.9 |
| Employee | 2 | 3.8 |
| **Residence location** |  |  |
| Rural | 47 | 88.7 |
| Urban | 6 | 11.3 |

1. **Clinical characteristics**

**Table 2.1. Maternal clinical characteristics**

| **Variables** | **No.** | **%** |
| --- | --- | --- |
| **Clinical presentation or complaint** |  |  |
| Epigastric pain | 22 | 41.5 |
| Dyspnea | 0 | 0.0 |
| Coma | 5 | 9.4 |
| Palpitation | 0 | 0.0 |
| Low limbs oedema | 18 | 34.0 |
| Headache | 34 | 64.2 |
| Blurred vision | 6 | 11.3 |
| Convulsions | 48 | 90.6 |
| **Obstetric history** |  |  |
| Nulliparous | 23 | 43.4 |
| Multiparous (≤4 births) | 25 | 47.2 |
| Grand-Multiparous (≥5 births) | 5 | 9.4 |
| **History of pregnancy loss** |  |  |
| Yes | 8 | 15.1 |
| No | 45 | 84.9 |
| **Gestational age** |  |  |
| ≤28week | 4 | 7.5 |
| 28-33 weeks | 21 | 39.6 |
| 34-37 weeks | 13 | 24.5 |
| 38-40 weeks | 10 | 18.9 |
| LMP unknown | 5 | 9.4 |
| **Gestational period at time of enrollment** |  |  |
| Antepartum | 0 | 0.0 |
| Intrapartum | 1 | 1.9 |
| Postpartum | 52 | 98.1 |
| **Number of intrauterine foetus** |  |  |
| Single intrauterine pregnancy | 53 | 100.0 |
| Twins pregnancy | 0 | 0.0 |
| **ANC visit attendance** |  |  |
| Yes | 49 | 92.5 |
| No | 4 | 7.5 |
| **Facility of ANC visit** |  |  |
| None | 4 | 7.5 |
| Teaching & Referral Hospital | 1 | 1.9 |
| County referral Hospital | 4 | 7.5 |
| Sub-county Hospital | 6 | 11.3 |
| Health centre | 26 | 49.1 |
| Private Hospital | 2 | 3.8 |
| Dispensary | 10 | 18.9 |

**Table 2.2. Maternal BPs and laboratory results**

| **Variables** | **No.** | **%** |
| --- | --- | --- |
| **Blood pressure (BPs)** |  |  |
| <140/90 mmHg | 7 | 13.2 |
| ≥140/90 mmHg | 46 | 86.8 |
| **Proteinuria** |  |  |
| Trace | 7 | 13.2 |
| 1+ | 8 | 15.1 |
| 3+ | 30 | 56.6 |
| 4+ | 8 | 15.1 |
| **Platelet (PLT)** |  |  |
| ≥150.10⁹/l | 42 | 79.2 |
| ≤100.10⁹/l | 6 | 11.3 |
| ≤50.10⁹/l | 5 | 9.4 |
| **Peripheral blood smear (PBS)** |  |  |
| Normal | 50 | 94.3 |
| Abnormal | 3 | 5.7 |
| **AST/ALT** |  |  |
| <70 IU/l | 47 | 88.7 |
| ≥70 IU/l | 6 | 11.3 |
| **Serum Bilirubin** |  |  |
| Normal | 49 | 92.5 |
| Increased | 4 | 7.5 |
| **L-lactate dehydrogenase (LDH)** |  |  |
| <600IU/l | 49 | 92.5 |
| ≥600IU/l | 4 | 7.5 |
| **Creatinine** |  |  |
| ≤80 mmol/l | 45 | 84.9 |
| >80 mmol/l | 8 | 15.1 |

AST: aspartate transaminase; ALT: alanine transaminase

**Table 2.3. Management**

| **Variables** | **No.** | **%** |
| --- | --- | --- |
| **Mode of admission** |  |  |
| From home | 24 | 45.3 |
| Transferred | 29 | 54.7 |
| **Treatment antepartum** |  |  |
| Yes | 10 | 18.9 |
| No | 43 | 81.1 |
| **Medications** |  |  |
| Anticonvulsants | 8 | 15.1 |
| Multiples drugs | 45 | 84.9 |
| **Mode of delivery** |  |  |
| Vaginal | 24 | 45.3 |
| Caesarean | 29 | 54.7 |
| Assistant vaginal delivery | 0 | 0.0 |
| **Indication of caesarean section** |  |  |
| N/A | 24 | 45.3 |
| Eclampsia | 26 | 49.1 |
| NRFS | 2 | 3.8 |
| Arrested disorders | 1 | 1.9 |
| **Labour onset** |  |  |
| None | 17 | 32.1 |
| Spontaneous | 27 | 50.9 |
| Cytotec | 4 | 7.5 |
| Induced Foley catheter | 1 | 1.9 |
| Catheter+ Cytotec | 4 | 7.5 |
| Oxytocin | 0 | 0.0 |

NRFS: non-reassuring foetal status
